# Oral ulcers and sarcoid-like reaction in lymph nodes after cemiplimab therapy for locally advanced cutaneous squamous cell carcinoma: a case report

**DOI:** 10.31744/einstein_journal/2022RC6367

**Published:** 2022-02-23

**Authors:** Mariana Henriques Ferreira, Letícia Mello Bezinelli, Fernanda de Paula Eduardo, Marcella Ferreira Gobbi, Luciana Corrêa, Gustavo Schvartsman

**Affiliations:** 1 Hospital Israelita Albert Einstein São Paulo SP Brazil Hospital Israelita Albert Einstein, São Paulo, SP, Brazil.; 2 Faculdade de Odontologia Universidade de São Paulo SP Brazil Faculdade de Odontologia, Universidade de São Paulo, SP, Brazil.

**Keywords:** Carcinoma, squamous cell, Oral ulcer, Skin neoplasms, Immunotherapy

## Abstract

Cemiplimab is a novel programmed death-1 inhibitor recently approved for advanced cutaneous squamous cell carcinoma. Immune-related adverse events derived from cemiplimab are similar to other anti-PD-1 drugs, including gastrointestinal and cutaneous toxicities. Oral immune-related adverse events were not reported with cemiplimab in previous studies; thus this case report warns of the fact that the oral cavity may be a site of immune-related adverse events during programmed death-1 block therapy and that this can lead to significant limitations when not properly treated. The present report describes the case of a patient with locally advanced cutaneous squamous cell carcinoma metastatic to cervical lymph nodes who developed dysphagia due to large and painful oral ulcers after a single dose of cemiplimab. The patient also exhibited a sarcoid-like reaction in mediastinal lymph nodes. No immune-related adverse events were found in any other organs. The oral lesions showed significant improvement after topical and short-course systemic corticosteroids, and low-level laser therapy was also performed in the oral lesions. The patient achieved a near-complete response and treatment was discontinued. This article discusses in detail the clinical outcomes and oral toxicity management of cemiplimab therapy for cutaneous squamous cell carcinoma.

## INTRODUCTION

Cemiplimab is a high-affinity human monoclonal antibody that binds to programmed death-1 (PD-1) receptors localized in activated T and B lymphocytes and macrophages, blocking the attachment of its ligands programmed death ligand-1 (PD-L1) and programmed death ligand-2 (PD-L2).^(1)^ Phase 1 and 2 trials have demonstrated promising results, with overall response rates of up to 47%, but at the cost of serious treatment-related adverse events in up to 29% of the patients, including cellulitis, pneumonitis, hypercalcemia, pleural effusion, and death.^(2,3)^

To the best of our knowledge, no adverse event in the upper digestive tract was ever reported with cemiplimab. This report describes a case of a patient with recurrent, locally advanced cutaneous squamous cell carcinoma (cSCC) metastatic to regional cervical lymph nodes and parotid gland treated with cemiplimab. The patient developed large and painful ulcerations in the oral and oropharyngeal mucosa that were presumably related to the drug. A sarcoid-like reaction in mediastinal lymph nodes was also detected in PET-CT scans.

## CASE REPORT

This is an 85-year-old white male patient with a past medical history of controlled hypertension and diabetes, who was diagnosed in 2016 with a cSCC in the right palpebral region. He underwent a complete surgical resection. Histopathological exam revealed a poorly differentiated cSCC, with 2.5cm of diameter, showing perineural infiltration and 1mm of depth of invasion. The surgical margins were negative, and he received no adjuvant therapy.

**Figure 1:**
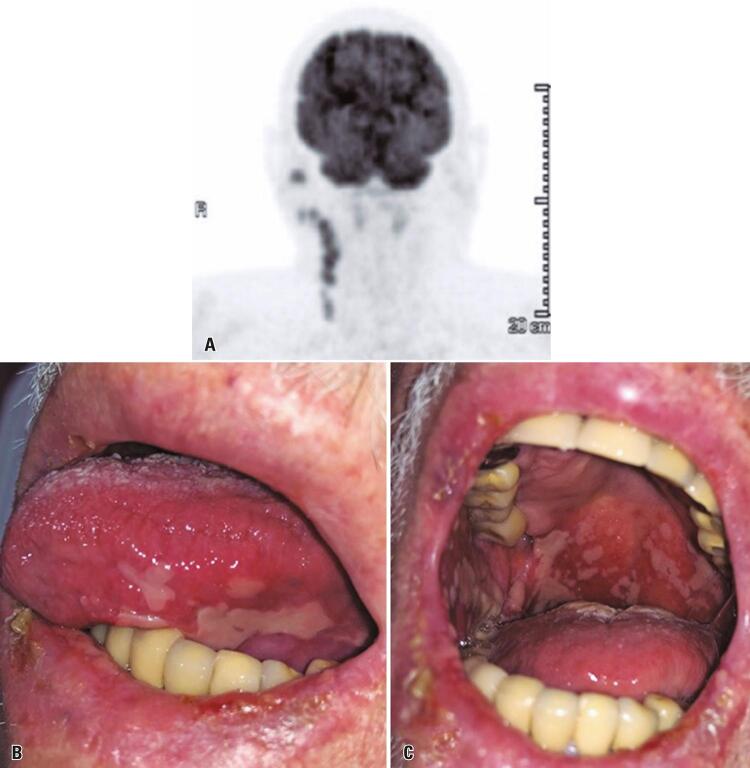
(A) baseline PET-CT showing FDG-avid right parotid and cervical lymph nodes. (B) and (C) clinical aspect of oral ulcers after two weeks of the first dose of cemiplimab. The lesions were extremely painful and the patient reported dysphagia

In December 2019, the patient noted a volume increase in the right parotid gland region, along with ipsilateral cervical lymph nodes. A PET-CT scan showed hypoattenuating nodular formations in the right parotid gland, invading both the superficial and deep lobes, measuring up to 1.6cm (SUVmax: 7.7; Figure 1A). Additionally, enlarged ipsilateral cervical lymph nodes of up to 2cm were also detected (SUVmax: 5.4). No distant metastases were found. Treatment plan with cemiplimab at 350mg, every three weeks was proposed and accepted after a risk-benefit discussion with the patient and his family.

On cycle 1, day 8 (C1D8), the patient reported a mild odynophagia, with scattered white exudates in the oropharynx. Oropharynx samples were negative for Group A *Streptococcus* in a rapid antigen test.

In C1D15, the pain in the oral cavity worsened. On the exam of oral mucosa, several ulcerative painful lesions were noted (Figure 1B and C). The lesions were large, coalescent, and covered by a pseudo-membrane, resembling a chemotherapy-associated oral mucositis or a drug-associated stomatitis. Skin manifestations were not seen on exam. Hemogram, renal and liver functions were within normal range. Dipyrone 1g q6h and ketorolac tromethamine at 10mg q6h p.r.n. were prescribed. Oral biopsies were performed in the lower lip and cheek mucosa. Histopathological analysis revealed a non-specific inflammatory reaction in the oral mucosa, composed by ulcerated epithelium covered by fibrin and leukocyte exudate. No sign of malignant cells, bacterial or fungal infection were noted. The histopathological diagnosis favored a drug-induced stomatitis. A topical treatment with dexamethasone elixir (0.1mg/mL, 10mL, three times a day, for 14 days) was prescribed, as well as oral prednisone at a dose of 80mg (1mg/kg). For pain relief, one daily session of low-level laser therapy using red and infrared lasers was performed in the oral lesions for four days (660nm and 808nm simultaneously, 100mW, 3J per point, 0,09cm^2^ spot area).

**Figure 2:**
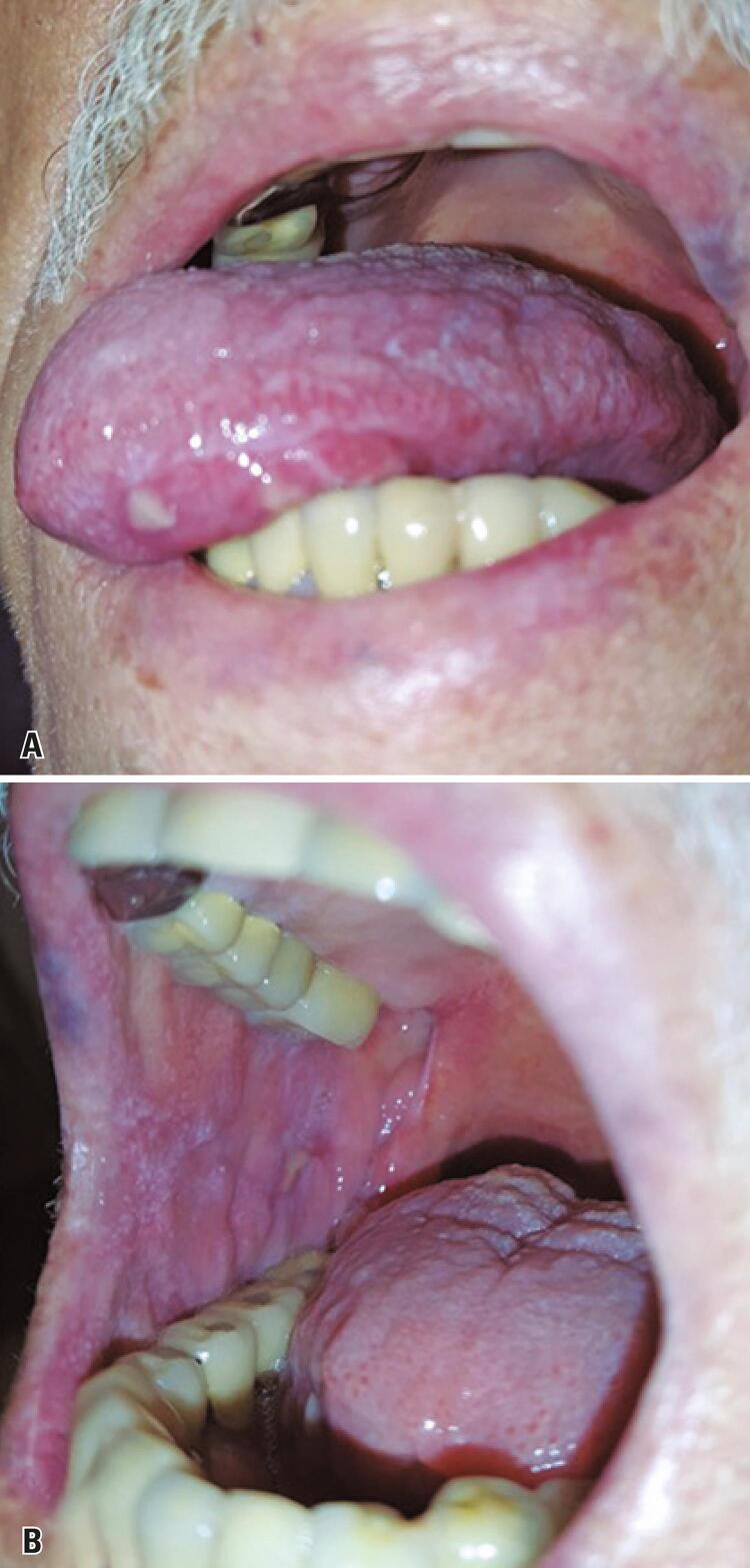
(A and B) significant improvement of the lesions, with advanced reepithelization of the ulcers and complete pain remission after treatment; the lesions were treated with topical and systemic corticosteroids and low-level laser therapy

From C1D16 to C1D20, the oral ulcers showed a significant clinical improvement, with high reepithelization degree (Figure 2A and B). Complete remission of the oral lesions occurred by C1D29.

**Figure 3:**
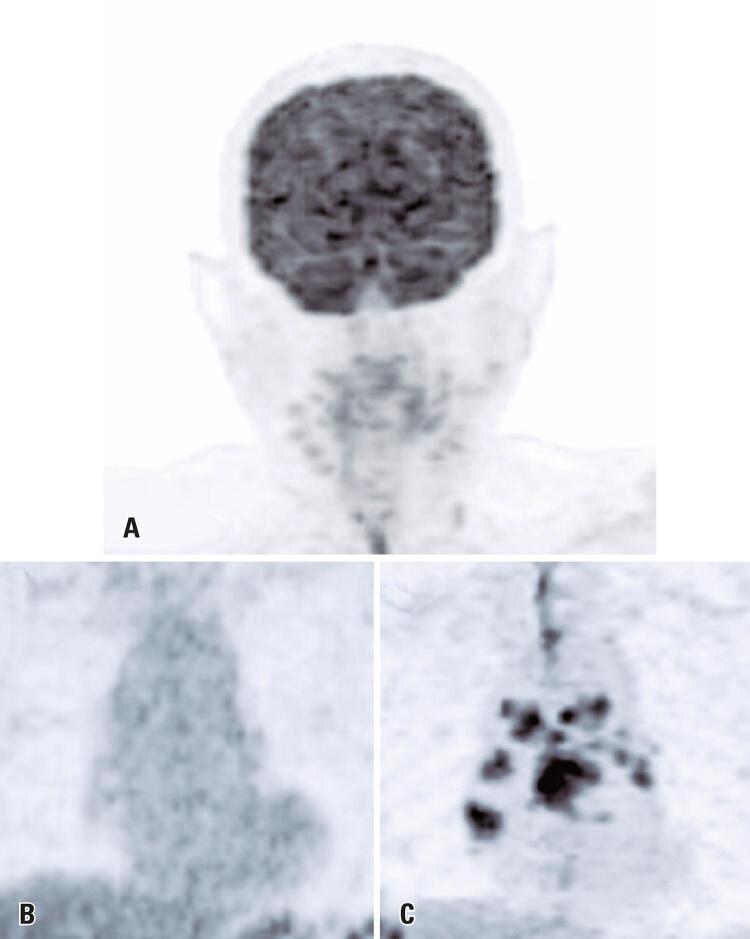
(A) PET-CT performed six weeks after one dose of cemiplimab showing an excellent partial response; (B) baseline PET-CT showing no mediastinal lymph nodes with FDG uptake; (C) PET-CT performed six weeks after one dose of cemiplimab showing a sarcoid-like reaction in the mediastinal lymph nodes

On C1D40, the repeat PET-CT showed a near-complete reduction in size and FDG uptake in the parotid gland and lymph nodes (SUVmax: 4,2; Figure 3A). Interestingly, a sarcoid-like reaction was detected in mediastinal lymph nodes (Figure 3B and C). In the histopathological analysis of the oral lesion, no granulomatous formation was found. Due to the favorable tumor response and the oral toxicity severity, cemiplimab therapy was discontinued. Currently (June 2021), the patient exhibits good systemic conditions, is disease-free, and not under any antineoplastic treatment.

This study was approved by the Research Ethics Committee of *Hospital Israelita Albert Einstein* under number # 4.616.775, CAAE: 44575021.3.0000.0071.

## DISCUSSION

Cemiplimab was the first systemic agent approved for metastatic and locally advanced cSCC, currently considered the standard first-line treatment. Phases I/II trials showed an overall response rate of 50%. Among patients who had a response, the duration exceeded six months in 57%, and 82% of responses were ongoing until data cutoff.^(2,3)^

In general, immune-related adverse events (irAEs) in the oral cavity associated to anti-PD-1/PD-L1 antibodies have been rarely reported in the literature. Lichenoid reactions in the oral mucosa, with striae, white patches and ulcers, as well as xerostomia, are described in patients treated with nivolumab, atezolizumab, and pembrolizumab.^(4-7)^ One study reported 13 patients who showed ulcerative and lichenoid (77.0%) lesions, erythema multiforme (15.3%), and reactivation of graft-*versus*-host disease (7.7%) in the oral cavity, which were associated to pembrolizumab (46.2%) or nivolumab (53.8%).^(8)^ In a brief review of the literature, 20 clinical reports showing cases of oral lesions associated to PD-1 blockades were found, but none of them was related to cemiplimab (Table 1). The most common clinical aspects were unspecific oral ulcers, and oral erosions compatible with pemphigoid lesions. The majority of these lesions were treated with topical corticosteroids, associated or not to systemic administration of these drugs. Some cases demanded discontinuation of the checkpoint inhibitors. In the present report, the oral lesions had high severity, but showed a rapid remission after topical and oral corticosteroid therapy, associated with low-level laser therapy. Other reports described the efficacy of low-level laser therapy for irAEs in the oral cavity.^(5,6)^

**Table 1:** Summary of clinical aspect, diagnosis, and treatment of oral lesions induced by PD-1 blockades reported in the literature*

PD1 blockade	Clinical aspect or diagnosis of the oral lesion	Treatment
Nivolumab	Bullous pemphigoid (n=3)	Tacrolimus ointment (n=1) Dexamethasone swish/spit (n=1) Oral corticosteroids (n=2) Rituximab (n=1) Drug discontinuation (n=2)
	Painful oral ulcers/erosions (n=5)	Topical hydrocortisone ointment (n=1) Topical triamcinolone acetonide (n=1) Oral corticosteroids (n=3) Drug discontinuation (n=4)
Pembrolizumab	Bullous pemphigoid (n=1)	Clobetasol ointment
	Paraneoplastic pemphigus (n=1)	Oral corticosteroids, drug discontinuation
	Lichen planus pemphigoid (n=1)	Topical clobetasol, oral prednisolone, rituximab, PUVA therapy, acitretin, sirolimus, dapsone
	Mucous membrane pemphigoid (n=3)	Doxycycline therapy (n=2) Mouthwash with betamethasone (n=2) Topical mometasone furoate (n=1) Oral corticosteroids (n=1) Mycophenolate mofetil (n=1) Methylprednisolone pulse therapy (n=1) Infliximab and rituximab (n=1) Intravenous immunoglobulin (n=1) Drug discontinuation (n=1)
	Oral ulcers (n=4)	Mouthwash with “magic solution”** (n=1) Dexamethasone swish/spit (n=1) Triamcinolone ointment (n=1) Oral corticosteroids (n=3) Intravenous methylprednisolone (n=1) Drug discontinuation (n=2)
	Steven-Johnson syndrome (n=2)	Cyclosporine (n=1) Oral corticosteroids (n=1) Drug discontinuation (n=1)

* A total of 20 clinical reports were selected considering the presence of an adequate description of the oral lesions and their treatment. The list of these references is disposable as supplementary documentation. The numbers in parenthesis indicate the frequency of studies.

** magic solution = nystatin, hydrocortisone, and diphenhydramine solution (n=1).

PD1: programmed death-1; PUVA: Psoralen Ultra-Violet A.

Besides the oral toxicity, the patient also showed a sarcoid-like reaction in the mediastinal lymph nodes, an irAE previously described during anti-PD-1 and anti-CTLA-4 therapies,^(9,10)^ such as pembrolizumab, nivolumab, and ipilimumab.

This report alerts that the oral cavity can be a site of irAEs during PD-1 blockade therapy and that these lesions can induce important limitations, such as dysphagia and significant pain. Prompt treatment initiation should be prioritized. This case also signalizes the potential of cemiplimab to induce non-infectious granulomatous reaction. Cemiplimab resumption should be discussed in a case-by-case situation based on the long-term efficacy characteristic of this class of drugs.
